# Patient-Centered Tuberculosis Programs Are Necessary to End the Epidemic

**DOI:** 10.1093/infdis/jix373

**Published:** 2017-11-06

**Authors:** Michael J A Reid, Eric Goosby

**Affiliations:** 1 Division of HIV, ID and Global Medicine, Department of Medicine, Zuckerberg San Francisco General Hospital and Trauma Center, and; 2 United Nations Secretary-General’s Special Envoy on Tuberculosis, San Francisco, California

**Keywords:** tuberculosis, patient-centered care, private sector engagement

Tuberculosis elimination can be achieved, but only if we are able to identify and successfully treat the 4 million individuals [1] with tuberculosis who are currently undiagnosed. The studies in this supplement clearly demonstrate that to do so we must address the reality that many of those undiagnosed patients are presenting for care at health facilities ill equipped to diagnose and/or treat tuberculosis. For example, in Pakistan, the Philippines, and Indonesia, most first contact care happens in the private sector. Unfortunately, the data presented in articles by authors R. K. Fatima (R.K. Fatima, M. Haq and N. Mahmood et al., submitted) and C. Garfin et al. (C. Garfin, M. Mantala, R. Yadav et al., submitted) reveal that the private sector in these countries includes a heterogeneous mix of services that are underequipped to diagnose and manage tuberculosis patients. We will simply not reach tuberculosis elimination without a renewed commitment to engaging the private sector in tuberculosis diagnosis and treatment efforts. Furthermore, we need a patient-centered approach that not only is able to diagnose tuberculosis but also gets the patient on treatment and monitors treatment progress. Uniformly, the studies speak to the need for national tuberculosis programs (NTPs) to urgently reconfigure how and where diagnostic resources are allocated to ensure patients with tuberculosis are diagnosed and treated promptly.

## MEETING THE PATIENTS WHERE THEY ARE

Across all of the countries presented in the supplement, the striking finding is the lack of tuberculosis diagnostic capacity at the health-sector venues where people are first presenting for care. In Ethiopia, the authors estimate that only 46% of first-contact patients are likely to visit a facility that has tuberculosis diagnosis services available (L. Fekadu, C. L. Hanson, M. Osberg et al., submitted); in Indonesia, the estimate is 24% (A. Surya, B. Setyaningsih, H. S. Nasution et al., submitted). Acknowledging that these figures may not account for facilities that receive diagnostic support from central laboratories, the findings still provide stark evidence of why diagnosing and treating tuberculosis is so challenging in these countries. Faced with no access to microbiologic or radiologic tools, many tuberculosis patients encounter long delays before starting antituberculous therapy or, worse still, never even start treatment. These delays are undoubtedly far longer, with worse clinical consequence, for individuals with drug-resistant disease and children, who often are difficult to diagnose and do not always exhibit the same symptoms as adults.

Nonetheless, these studies provide useful insights for NTPs to address those delays and improve the allocative of their programs. By identifying the provinces and districts with the biggest mismatches between care-seeking and diagnostic capacity, these data provide powerful levers to inform strategic planning and budgetary decisions. They can also be used to improve the allocative efficiency of donor investment, guiding funding at subnational and district levels and across socioeconomic and urban–rural subpopulations. National tuberculosis programs in several countries have already used the patient pathway analysis as a tool to inform national strategic planning and guide donor funding requests to close gaps in service provision (C.L. Hanson, M. Osberg, J. Brown et al., submitted). It will be important to assess the impact of this approach, not only to optimize resources but also to drive quality improvement along the care continuum to ultimately reduce tuberculosis incidence and save more lives.

## STRENGTHENING PRIVATE–PUBLIC MIX

Several of the studies in the supplement persuasively illustrate that there is a substantial burden of tuberculosis care-seeking behavior that happens outside of the public health system. In Pakistan and Indonesia, for example, the authors (R.K. Fatima, M. Haq and N. Mahmood et al., submitted; A. Surya, B. Setyaningsih, H. S. Nasution et al., submitted) demonstrate that 53% and 71% of care-seeking behavior occurs in the private sector; in Kenya 15% of first contact is in informal settings (E. Masini, C. L. Hanson, J. Ogoro et al., submitted). These findings confirm what other studies have clearly demonstrated: a substantial amount of first-contact care happens in the private sector and/or is provided by pharmacists and traditional healers [2–5]. Unfortunately, these providers are far less likely to have the diagnostic capacity to detect tuberculosis, let alone treat it adequately. Indeed, lack of diagnostic capacity in the private sector is part of the reason why there are often substantial delays in case identification and unnecessary expenditure for patients [6].

Nevertheless, in view of the dominant role that private healthcare providers play in tuberculosis care, strengthening linkages between the private sector and public tuberculosis clinics, as well as connecting private providers to national notification systems, is crucial to achieve tuberculosis control targets [7]. National tuberculosis programs need to engage with the private sector in the formulation of strategic plans to streamline access to diagnostic capabilities in the public sector and/or develop business models that incentivize private practitioners to invest in tuberculosis diagnostic resources. The impact of scaling up access to Xpert MTB/RIF in the public sector in countries like Pakistan and Indonesia will be extremely muted unless there are concomitant efforts to ensure that these tools are available to private providers [8]. The patient pathway analysis outlined in this supplement is one way to help draw attention to how discrepant tuberculosis diagnostic capability is in the private sector. It can and should be used as a powerful advocacy tool to engage private providers and to develop better models of care to ensure these providers have the resources and expertise to diagnose patients with tuberculosis disease.

## BETTER FRONT-LINE DIAGNOSTICS STILL NEEDED

Implicit in the conclusions of all of the studies included here is the need for more global investment in developing accurate, affordable, point-of-care diagnostics that can be used by frontline providers. Roughly 60% [9] of patients with tuberculosis worldwide present in peripheral health centers in resource-constrained settings where diagnostic tests based on sophisticated instruments such as Xpert MTB (Mycobacterium tuberculosis) / RIF (Rifampicin) are not feasible. Many of these facilities do not have access to running water or electricity. Therefore, an urgent challenge remains to ensure that new diagnostic tests are suitable for use in peripheral health facilities. Although the GeneXpert Omni platform (Cepheid, Sunnyvale, CA) is a step in the right direction, its expense and infrastructure requirements may limit its reach into the private sector and at the lowest levels of the public health system in many countries. The research and development community must remain focused on delivery of better point-of-care diagnostic tests that can be deployed even in the most rural health posts.

## SUMMARY

The kind of patient pathway analysis described in this supplement illustrates how NTPs can and should be responsive to the needs of their constituents: the patients with tuberculosis. This patient-centered approach to reconfiguring tuberculosis resources can also be a catalyst to improve private-sector engagement. By identifying gaps in tuberculosis diagnostic capacity at provincial and district levels, a framework can be established to redirect resources and funding to ensure that front-line providers have the resources to diagnose and treat the 4 million missing patients.

## Notes

***Disclaimer.*** The contents of this publication are solely the responsibility of the authors.

***Financial support.*** M. J. A. R. received funding support from the University of California San Francisco Traineeship in AIDS Prevention Studies (US National Institutes of Health T32 MH-19105).

***Supplement sponsorship.*** This work is part of a supplement sponsored by the Bill & Melinda Gates Foundation.

***Potential conflicts of interest.*** Both authors: No reported conflicts of interest. Both authors have submitted the ICMJE Form for Disclosure of Potential Conflicts of Interest. Conflicts that the editors consider relevant to the content of the manuscript have been disclosed.
